# Modern Medical Miracle: Matched Unrelated Donor Hematopoietic Stem Cell Transplant After Aplastic Anemia

**DOI:** 10.7759/cureus.13050

**Published:** 2021-02-01

**Authors:** Emily Van Antwerp, Zachary A Koenig, Ryan McCarthy

**Affiliations:** 1 Department of Medicine, West Virginia School of Osteopathic Medicine, Lewisburg, USA; 2 School of Medicine, West Virginia University, Morgantown, USA; 3 Internal Medicine, West Virginia University, Martinsburg, USA

**Keywords:** aplastic anemia, bone marrow transplant, matched unrelated donor

## Abstract

Aplastic anemia is a hematological disease with deadly complications related to pancytopenia if not treated in a timely manner. First-line treatment consists of immunosuppressive therapy or matched sibling donor (MSD) hematopoietic stem cell transplant. Step up treatment involves a matched unrelated donor (MUD) hematopoietic stem cell transplant (HSCT) alongside immunosuppressant conditioning. However, recent research suggests that there is improved success of MUD HSCT for severe aplastic anemia compared to immunosuppressive therapy. We present a case of an 18-year-old who was diagnosed with severe aplastic anemia who received numerous immunosuppressive therapy regimens prior to obtaining a MUD HSCT. Over a year after bone marrow transplant, the patient is doing well with no signs of rejection. This case creates an argument for the use of upfront MUD HSCT as a curative treatment for acquired aplastic anemia rather than initial treatment with immunosuppressive agents.

## Introduction

Aplastic anemia is a rare, life-threatening condition that produces pancytopenia. The estimated incidence is two per million people per year, and the incidence is two to three times higher in Asia. There are no differences in aplastic anemia when stratified by sex. Individuals of all ages may be affected, although half who have acquired aplastic anemia occur before thirty years of age [1].

The defining characteristic of aplastic anemia is loss of hematopoietic stem cells and consequently failure to produce mature cells in peripheral blood and tissues. Normally, these cells are multipotent yet remain in a dormant proliferative state. Although the progenitor cells have the ability to self-renew, they can give rise to progenitor cells with a narrow lineage potential and high mitotic capacity. However, in aplastic anemia, cytotoxic T lymphocytes and type I cytokines mount an immune response against hematopoietic progenitor cells and destroy their ability to differentiate [2].

Lack of mature hematopoietic stem cells leads to an array of clinic manifestations. Neutropenia predisposes to recurrent infections. Thrombocytopenia leads to menorrhagia or mucosal hemorrhage. Progressive anemia causes pallor, exertional dyspnea, bounding pulses, tachycardia, and a flow murmur. The etiologies of aplastic anemia are wide ranging and include autoimmune mechanisms, direct injury to hematopoietic stem cells, viral infection, and clonal disorders. However, most cases are idiopathic but are believed to have a component of autoimmune destruction of hematopoietic stem cells [3].

Aplastic anemia requires prompt recognition and urgent treatment as it can be rapidly fatal. Initial workup should include a complete blood count, but a bone marrow biopsy establishes the diagnosis. A diagnostic bone marrow biopsy shows hypoplastic bone marrow (<50% normal cellularity with < 30% of the cells hematopoietic) in the absence of bone marrow infiltration with malignant cells or fibrosis. The residual hematopoietic stem cells appear normal and non-megaloblastic on microscopy [3,4]. Additional testing is needed to rule out secondary causes. 

The treatment of aplastic anemia depends on the age and severity of disease. If a human leukocyte antigen (HLA)-matched sibling is not initially available, immunosuppressive therapy and eltrombopag should be started. If immunosuppressive therapy fails, children can undergo alternative hematopoietic stem-cell transplantation and whereas adults typically undergo a second immunosuppressive therapy course. Failure of the second immunosuppressive therapy in adults is an indication for a matched unrelated donor or haploidentical bone marrow transplantation [5].

## Case presentation

In January 2018, an 18-year-old Caucasian man with a past medical history of asthma and hearing loss presented to his primary care physician with a chief complaint of syncope that was concurrent with vomiting, dizziness, tunnel vision, tinnitus, palpitations and shortness of breath. A new systolic ejection murmur was present on exam.

A week later, he presented to his local rural emergency department where he reported constant sleepiness and worsening shortness of breath despite stable vital signs. He underwent echocardiography which revealed mild aortic valve regurgitation in a morphologically normal, tri-leaflet and noncyanotic aortic valve. Bloodwork showed a white blood cell count of leukopenia (2.9 x 103/uL), anemia (3.4 g/dL), and thrombocytopenia (14,000 x 103/uL). He was subsequently admitted to the hospital and received 4 units of packed red blood cells and 1 unit of platelets. Hematology consultation recommended a bone marrow biopsy, which showed 5% cellularity, no blasts, decreased trilineage hematopoiesis, positive iron stores, and no increase in fibrosis. This led to a diagnosis of aplastic anemia.

Physicians worked promptly to determine the cause of his aplastic anemia. Genetic testing for 40 genes associated with hereditary bone marrow failure syndromes was negative. He was tested for hepatitis, Epstein-Barr virus, cytomegalovirus, parvovirus B19, and HIV, all of which were negative. His medication history was negative for any drugs associated with bone marrow toxicity. He had never used tobacco products or drank alcohol. He denied any exposure to radiation or toxins. He has no family or personal history of autoimmune conditions. He does, however, have a family history of aplastic anemia in his maternal grandmother and uncle. 

The patient was subsequently transferred to a tertiary care facility’s pediatric intensive care unit for continued treatment after he developed neutropenic fever. No organisms were identified, but he responded to vancomycin, metronidazole, and cefepime. He began combination horse anti-thymocyte globulin and cyclosporine, which he received for the following six months. During immune therapy, the patient suffered from common colds, pneumonia, and a case of influenza A. 

In September 2018, he began treatment with eltrombopag, and a potential bone marrow transplant donor was explored. In May 2019, the patient received a matched unrelated donor (MUD) hematopoietic stem cell transplantation (HSCT) with cyclophosphamide and anti-thymocyte globulin conditioning. The received product was 2.0 X 108 nucleated cells/kg TNC and 2.0 x 106 CD34+ cells. His post-transplant course was complicated by hemorrhagic cystitis which improved after bladder irrigation. 

**Figure 1:**
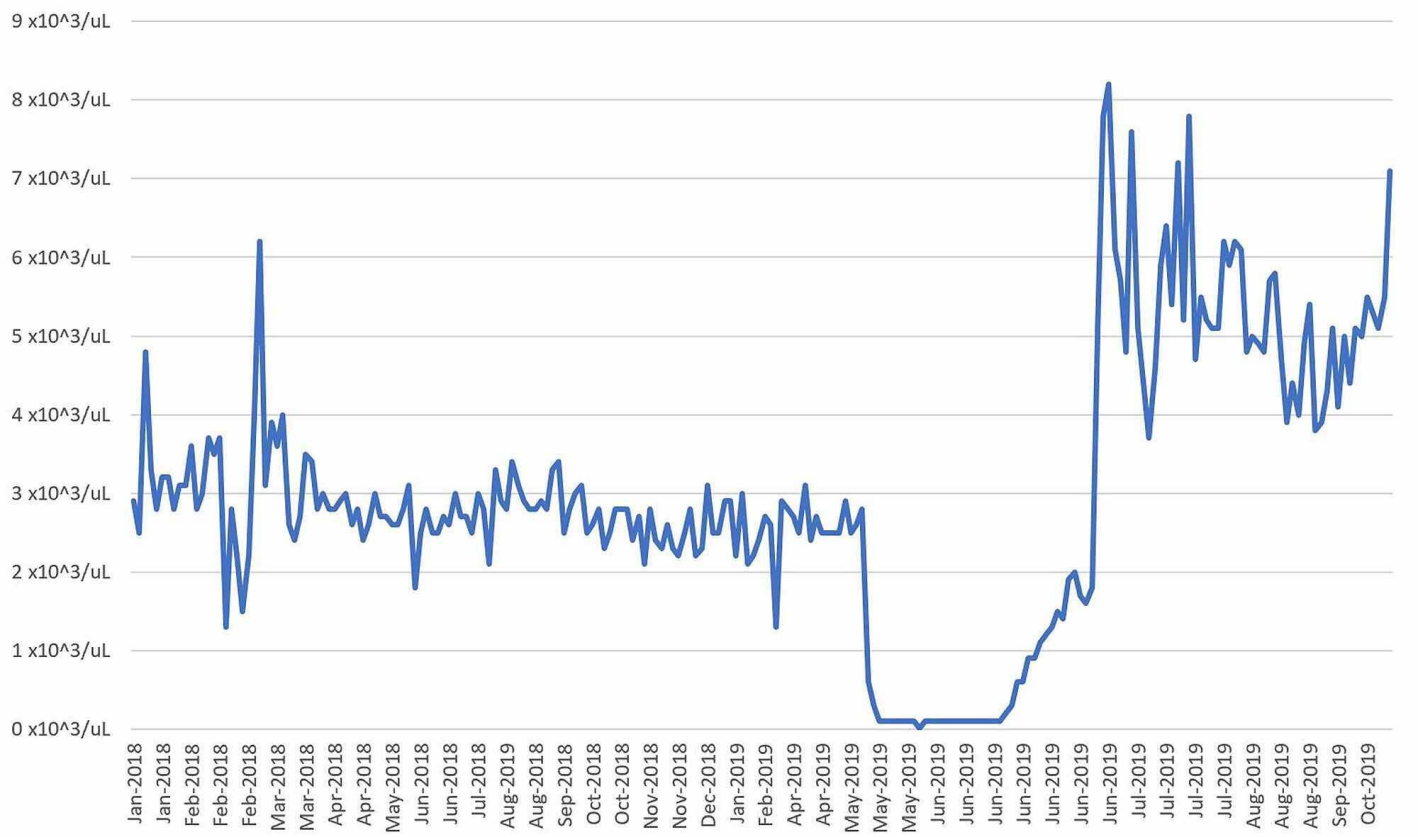
White Blood Cells vs Date

**Figure 2:**
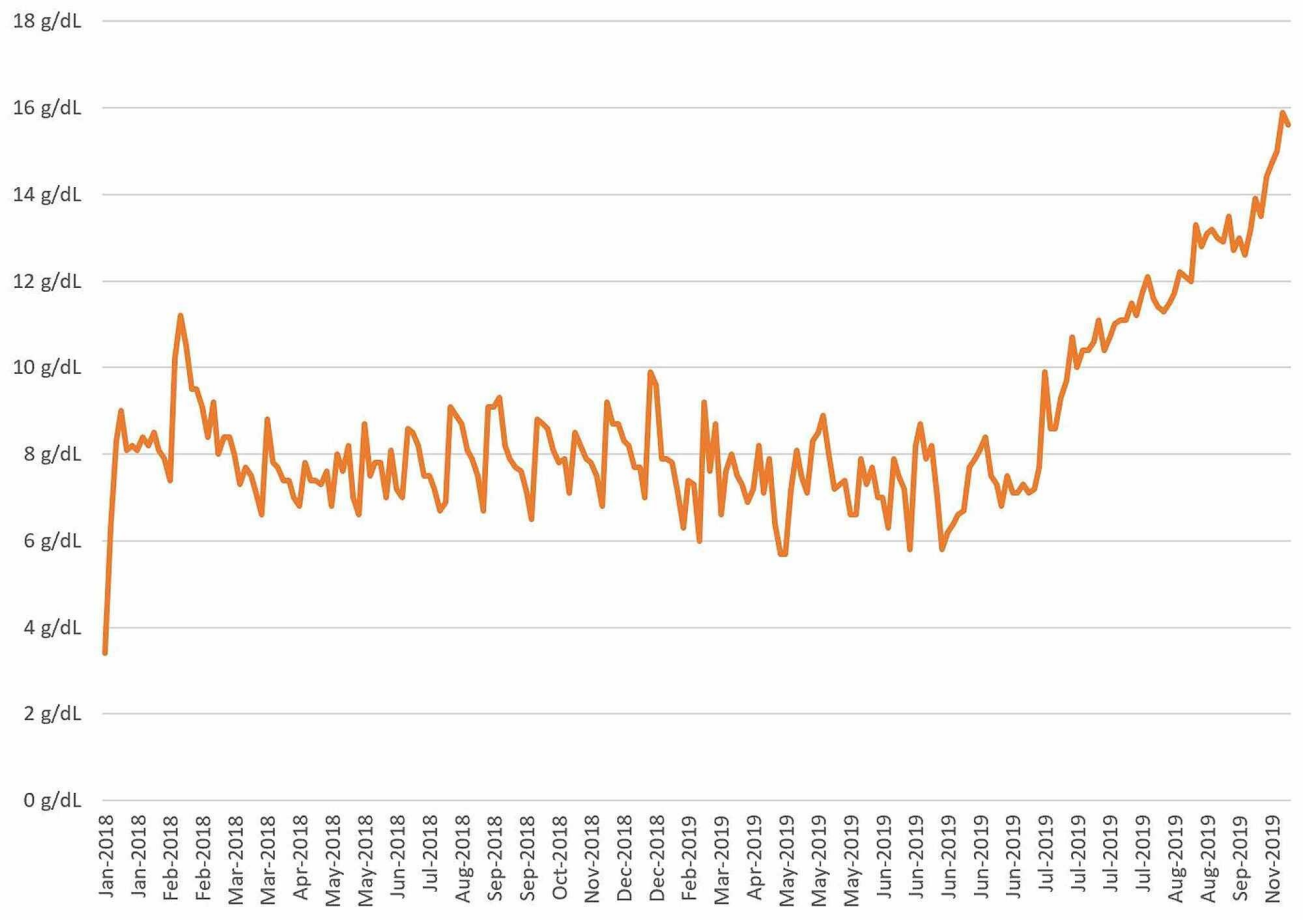
Hemoglobin vs Date

**Figure 3:**
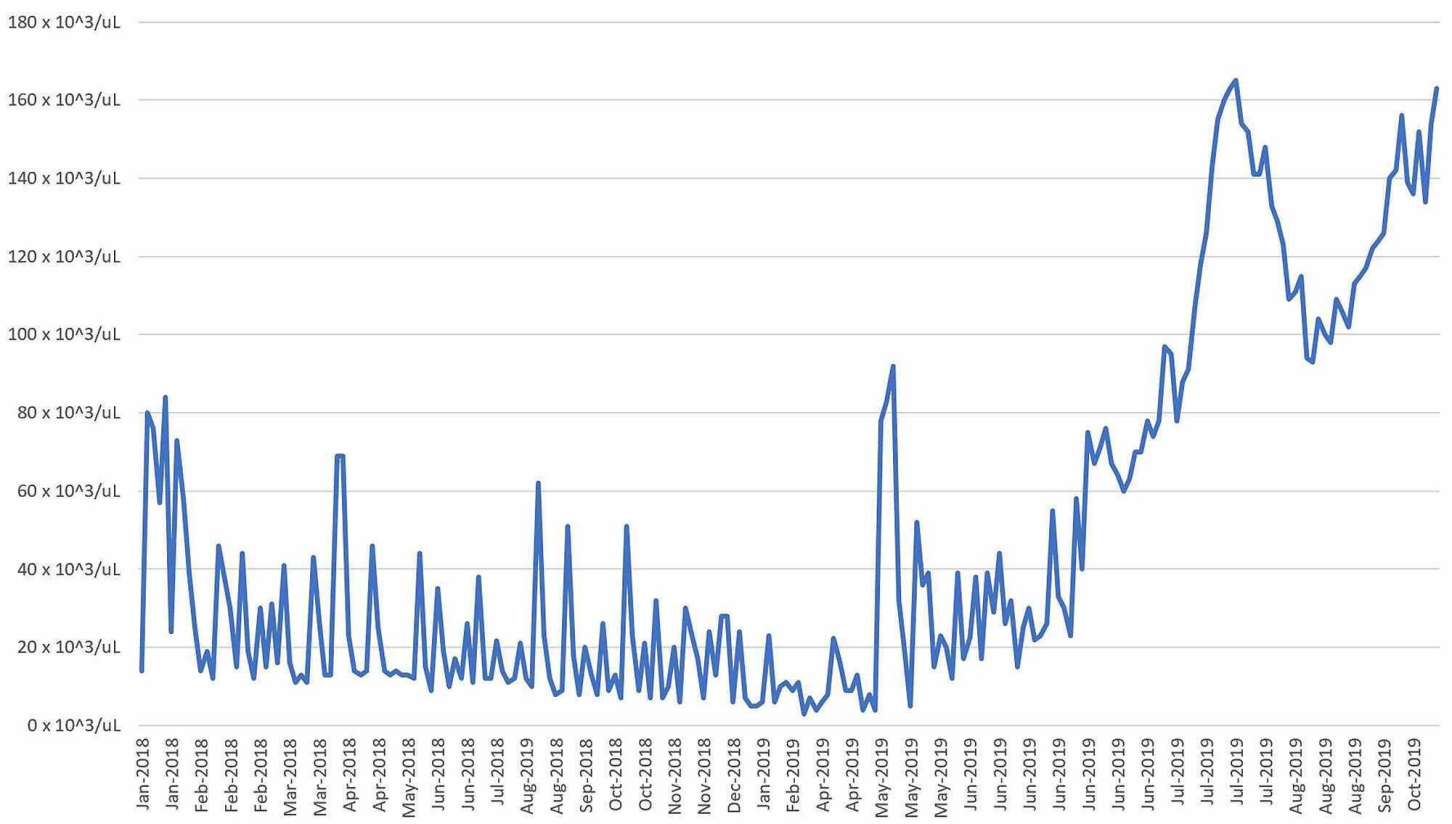
Platelet Count vs Date

Twenty-seven days after transplant the patient was discharged home with prophylactic acyclovir, fluconazole, tacrolimus, and letermovir. His cell lines have improved immensely within one month of the bone marrow transplant, leading to complete resolution of his prior pancytopenia (to date leukocyte count: 4,72 x 103/uL; platelet count: 156 x 103/uL; hemoglobin: 13.9 g/dL). His white blood cell count, platelet count, and hemoglobin were trended to ensure that he was exhibiting a therapeutic response to the bone marrow transplant (Figure 1, 2, 3).

Over a year has passed since his bone marrow transplant. He was followed up with at his rural hematology clinic in September 2020 where he has remained refractory to his previous aplastic anemia. He showed no signs of graft failure, graft-versus-host disease, or cancer. His complete blood count has remained stable, and he stated that he has been able to return to the life he once had. 

## Discussion

Acquired aplastic anemia continues to be a diagnosis of exclusion, which was the means behind reaching our patient’s diagnosis after all genetic, pharmacological, environmental, and infectious causes of aplastic anemia were ruled out. The treatment plan devised for our patient was consistent with the one set forth in an article in the New England Journal of Medicine [5]. Because only 22% of children with aplastic anemia have an HLA-matched sibling, immunosuppressive therapy is the preferred option. The initial regimen frequently consists of antithymocyte globulin alone or cyclosporine alone which has an estimated response rate of 50% [6]. However, combination immunosuppressant therapy has a response rate of 77% [6,7]. Several case series proposed that these response rates to combined immunosuppressant therapy were superior in pediatric patients compared to adults; however, our pediatric patient did not exhibit a clinical response to combination therapy [8,9]. For immunosuppressant refractory aplastic anemia, recommended management in young adults is bone marrow transplant from a matched unrelated donor or umbilical cord blood [5,7]. 

An HLA-matched sibling donor is the person of choice in HSCT for aplastic anemia. When this option is not feasible, the next best donor is a MUD HSCT. The more similar the HLA sequences are between the donor and the recipient, the more likely MUD HSCT is to be successful. With over 5500 class I alleles and over 1600 class II alleles, there are several million potential HLA combinations, which complicates the matching process [10]. Additionally, the polymorphic nature of HLA and abundance of haplotypes in African Americans and Asian Americans further complicates this process. The chance of finding a matched unrelated donor for a Caucasian is much more likely than a minority in the United States due to high representation in HLA databanks, which benefited our patient in finding a suitable donor [11]. HLA-based algorithms can be used to predict the likelihood of finding a donor. Although not a primary contributor to the matching process, major histocompatibility complex (MHC)-encoded class I chain-related genes should also be as similar as possible to reduce the risk of complications [12]. Once the donor is cleared as suitable, the bone marrow or peripheral blood progenitor cells are harvested and prepared for bone marrow conditioning and subsequent transplantation.

Although HSCT can produce miracle outcomes, its potential for adverse effects must be significantly weighed. The primary complications associated with allogeneic hematopoietic cell transplantation (HCT) secondary to aplastic anemia include graft failure or rejection, graft-versus-host disease, and late malignancy. The risk of graft rejection or failure is increased in patients of older age and in those who produced a poor response to immunosuppressive therapy, such as our patient. Patients who present with graft rejection or failure are left with limited options for treatment, including possible re-transplantation. The incidence of this complication ranges from 10-20% in transplantation patients but the risk can be reduced by more aggressive treatment regimens, use of immunosuppression, and changes in the quality and quantity of preceding blood transfusion products [13]. Graft-versus-host disease (GVHD) occurs when the non-identical donor cells recognize the recipient as foreign and stimulates an immune reaction in the transplant patient. Risk factors associated with the development of GVHD include HLA mismatches, transplantation with unrelated donors, donor and recipient gender disparity, and the source of the graft. The exact incidence of GVHD in allogeneic HCT is unclear, with reported incidence ranging from 9-50% in patients who received an HCT from an HLA-identical sibling [13]. Nonetheless, the incidence, severity, and mortality from acute GVHD can be improved with prophylaxis consisting of methotrexate and cyclosporine [14]. Squamous cell cancer is the most common late malignancy reported after an allogeneic HCT in aplastic anemia patients with a peak incidence between five and 20 years post-transplant. Other complications seen with HCT in aplastic anemia patients include cataracts, short stature, cardiovascular disease, endocrine problems, and iron overload [14]. 

Despite the possible complications of HSCT, survival of aplastic anemia patients post-HSCT has been improving over time. One study demonstrated that overall survival improved from approximately 45% in the 1970s to approximately 90% during the late 1980s and 1990s [15]. Another report from the Japanese Hematopoietic Cell Transplantation Registry demonstrated that 10-year overall survival for a cohort of 329 children with severe aplastic anemia who underwent MUD HSCT was 90% and event-free survival was 86%. Cumulative incidences of late malignancy were extremely low, being 0.8% at 10 years follow-up and 2.5% at 20 years follow-up. Another encouraging observation was that approximately 83% of patients had returned to school or work at two years post-transplant, and this number increased to 90% by 20 years. Patients considered themselves to have an excellent quality of life with only mild residual symptoms, and the majority of those surviving beyond two years returned to a fully functional life [16]. 

## Conclusions

HSCT is the only curative therapy for aplastic anemia, although it has some serious complications. Increased time to transplant is associated with worse patient outcomes and therefore this process should be expedited as fast as possible. Although current guidelines recommend two trials of immunosuppressive therapy with a six-month window, our patient may have benefitted more from early matched unrelated donor hematopoietic stem cell transplantation and no immunosuppressive therapy. Future research should focus on investigating use of HSCT as a first-line treatment for aplastic anemia, even if the donor is unrelated to the recipient.
